# Comparison of fixed- and mobile-bearing total knee arthroplasty with a mean five-year follow-up: A meta-analysis

**DOI:** 10.3892/etm.2013.1122

**Published:** 2013-05-17

**Authors:** MENGQI CHENG, DESHENG CHEN, YONGYUAN GUO, CHEN ZHU, XIANLONG ZHANG

**Affiliations:** 1 Department of Orthopedics, The Sixth Affiliated People’s Hospital, Medical School of Shanghai Jiaotong University, Shanghai 200233;; 2 Department of Orthopedics, The General Hospital of Ningxia Medical University, Yinchuan, Ningxia 750004, P.R. China

**Keywords:** total knee arthroplasty, fixed-bearing, mobile-bearing, meta-analysis

## Abstract

Controversy exists regarding the clinical and radiological differences in outcomes between fixed-bearing (FB) and mobile-bearing (MB) total knee arthroplasties (TKAs) at the mid- or long-term follow-up. We therefore conducted a meta-analysis and systematic review of randomized controlled trials (RCTs) that have evaluated FB and MB TKAs. We searched the Cochrane Central Register of Controlled Trials (CENTRAL) in the Cochrane Library, Medline and Embase. The data, including demographic information, methodological quality, duration of follow-up, clinical and radiographical outcomes, patient preferences and complications, were extracted. The methodological quality of the studies was assessed in accordance with the guidelines presented in the Cochrane Handbook for Systematic Reviews of Interventions. Nine trials, studying 1,821 knees, were eligible for data extraction and meta-analysis. The Knee Society score and the maximum knee flexion demonstrated no difference between the FB and MB groups (P=0.47 and P=0.72, respectively). Similarly, no difference was revealed between the groups for radiological outcomes or general health results. An increased number of high-quality RCTs with long-term follow-ups are required to validate the results.

## Introduction

Total knee arthroplasty (TKA) has become a successful procedure for treating end-stage osteoarthritis and rheumatoid arthritis (1–5). The mobile-bearing (MB) TKA design is assumed to provide a greater freedom of motion compared with the fixed-bearing (FB) variant, since the insert does not restrict the natural movements of the femoral component. This enables the reproduction of tibial internal rotation during flexion, reduces contact stresses and linear wear of the polyethylene, and improves patellofemoral tracking (6–10). This may, in turn, have a positive effect on the fixation of the prosthesis to the bone, and thereby reduce the risk of a loosening of the implant occurring.

Numerous trials have focused on the comparison of clinical outcomes between MB and FB TKAs. During the short-term follow-up period, no significant difference in clinical improvement has been demonstrated between MB- and FB-implant groups (11–16). In a prospective study, Hanusch *et al* (15) demonstrated that there was no significant difference in the mean range of motion (ROM) or Knee Society score (KSS) between MB and FB implants, at a mean follow-up of 13.4 months. However, short-term results are not always indicative of mid- and long-term outcomes. Controversy exists with respect to the clinical and radiological differences in outcomes between FB and MB TKAs during the mid- to long-term follow-up. Matsuda *et al* (17) revealed that no differences were exhibited in the rotational alignment or the ROM between MB and FB implants for a total of 61 knee arthroplasties in the mid-term follow-up. By contrast, a study by Higuchi *et al* (18), of 76 TKAs, demonstrated that the postoperative-extension ROM was significantly improved following TKA using an MB implant, in comparison with that employing an FB implant, at the four-year follow-up.

The mid- to long-term results of the prosthesis are important in evaluating the efficiency of implant. Although several meta-analyses concerning the clinical differences between FB and MB TKAs have been performed (19–22), the pooled data were not analyzed according to the different follow-up periods. In addition, a number of new randomized controlled trials (RCTs) have recently emerged. Therefore, there is a requirement for a new systematic review to compare the clinical, radiological and general health results of the two types of prostheses, at the mid- to long-term follow-up period.

## Methods

### 

#### Search strategy

We searched the Cochrane Central Register of Controlled Trials (CENTRAL) in the Cochrane Library, Medline and Embase. The search strategies used are demonstrated in Table I, along with the time span for the searches. The bibliography of eligible studies was also searched to identify further relevant trials.

#### Eligibility criteria

The inclusion criteria for the articles selected were as follows: i) the studies involved adult patients who had undergone primary TKA; ii) the studies were RCTs comparing MB- and FB TKAs and iii) the mean follow-up was >5 years. Animal and cadaver studies were excluded, and no language restriction was used.

#### Data extraction

Data were extracted independently from the included studies by two reviewers (MC/DC), and disagreements were resolved through consensus. Relevant data included demographic information, methodological quality, duration of follow-up, clinical and radiographical outcomes, patient preferences and complications. Whenever studies pertained to the same population at different follow-up periods, the investigation with the longer duration of follow-up was retained to avoid the duplication of information. Authors were contacted to request any unclear or missing data. Disagreements concerning paper eligibility were resolved through discussion.

#### Assessment of methodological quality

The methodological quality of the trials was evaluated independently by two reviewers (MC/DC), without masking the trial names. The reviewers followed the instructions provided in the Cochrane Handbook for Systematic Reviews of Interventions (23). The following domains were assessed: sequence generation, allocation concealment, blinding, incomplete data outcomes, revealing of selective outcomes and any remaining biases. When the information in the study was inadequate, attempts were made to contact the authors in order to ensure that the study was evaluated correctly.

#### Data analysis

The meta-analysis was conducted in accordance with the recommendations of the Cochrane Collaboration, using Review Manager (RevMan) software, version 5 (Copenhagen: The Nordic Cochrane Centre, The Cochrane Collaboration, 2008). For continuous outcomes, the weighted mean difference (WMD) and the 95% confidence interval (CI) were calculated. For dichotomous outcomes, the relative ratio (RR) and the 95% CI were calculated. Heterogeneity was explored using the χ^2^ test and the I^2^ statistic. Heterogeneity was considered to be significant when the P-value from the χ^2^ test was <0.10, or when the I^2^ statistic was >50%. A fixed-effect model was used if there was no statistical evidence of heterogeneity; otherwise, a random-effect model was selected. The analysis was conducted on an intention-to-treat bias, whenever possible, and a funnel plot was used to explore the publication bias. P<0.05 was considered to indicate a statistically significant difference.

## Results

### 

#### Description of studies

**Figure 1.**
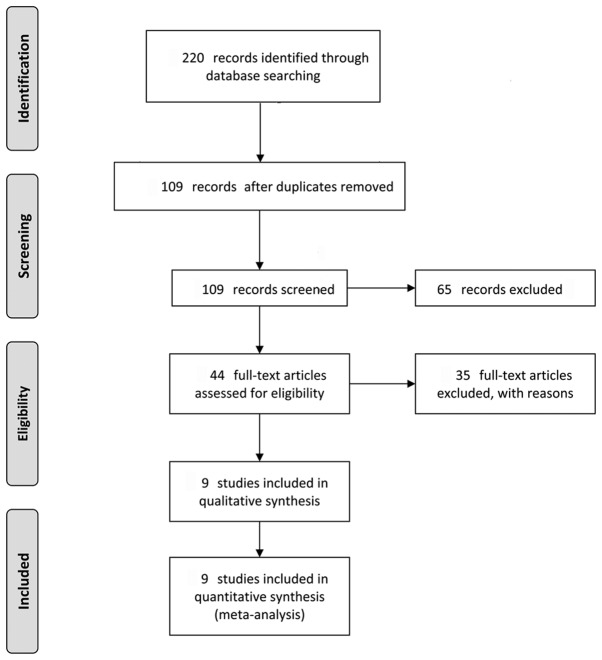
Preferred reporting items for systematic reviews and meta-analyses (PRISMA) flow diagram outlining literature search results.

A preferred reporting items for systematic reviews and meta-analyses (PRISMA) flow diagram outlining the literature search results is summarized in Fig. 1. In the initial search, a total of 220 references were identified electronically (Embase, 75; Medline, 92 and CENTRAL, 53). Following the application of the inclusion and exclusion criteria to the study titles and abstracts, only nine trials fulfilled the inclusion criteria.

#### Risk of bias in included studies

**Figure 2.**
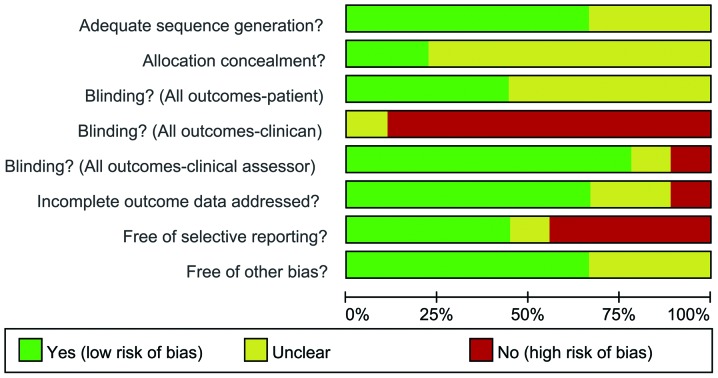
Risk of bias graph: a review of the authors’ judgments regarding each risk of bias item, presented as percentages across all included studies.

**Figure 3.**
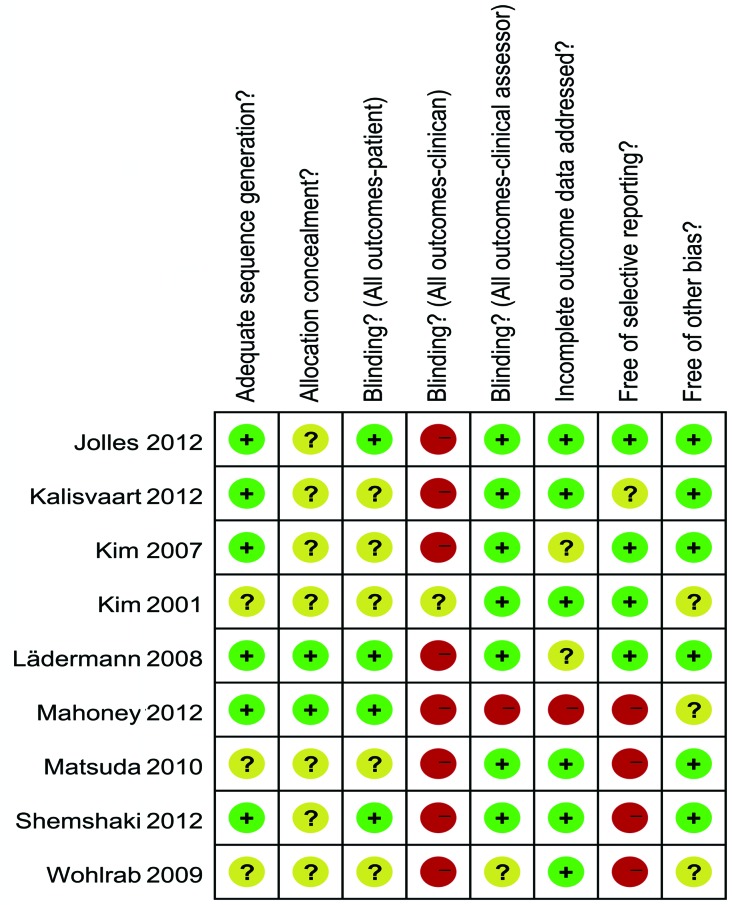
Risk of bias summary: a review of the authors’ judgments regarding each risk of bias item, for each included study.

**Figure 4.**
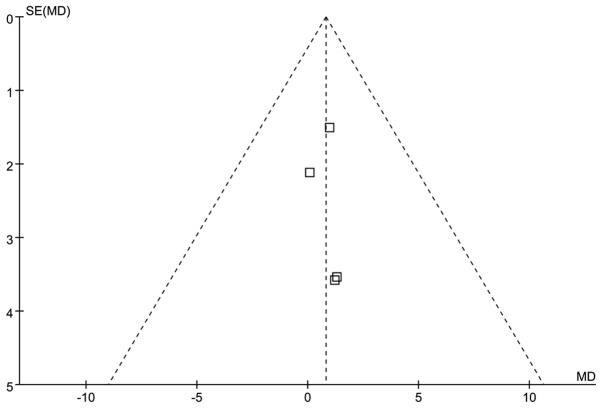
Funnel plot for the studies that utilized the Knee Society score (KSS).

The risk of bias is demonstrated graphically (Fig. 2) and also summarized (Fig. 3). Six (24–29) of the nine trials (66.7%) exhibited adequate sequence generation in the randomization process. However, only two trials (24,25) stated the method of allocation concealment. In the study by Lädermann *et al* (25), the allocation concealment was performed using sealed opaque envelopes, which were opened within 24 h prior to surgery; whereas in the study by Mahoney *et al* (24), allocation concealment was ensured by centralized randomization. Four of the nine trials (24–26,29) performed patient blinding. Clinician blinding of the implant type was impossible. All studies demonstrated assessor blinding, with the exception of two trials (24,30). A funnel plot analysis of the combined trials indicated a symmetry, demonstrating minimal recording bias (Fig. 4).

### Effects of intervention

#### Knee score

**Figure 5.**
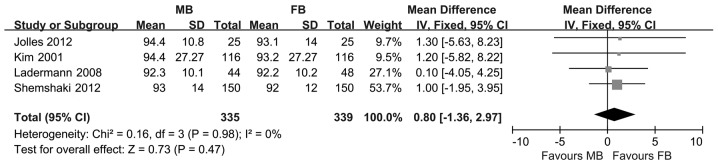
Forest plot comparing the Knee Society scores of mobile-bearing (MB) and fixed-bearing (FB) implants. CI, confidence interval.

The KSS (25,26,29,31), the Hospital for Special Surgery (HSS) knee score (30,31) and the Western Ontario and McMaster Universities Osteoarthritis Index (WOMAC) (26) were used for the clinical assessment of the patients (in four, two and one trials, respectively). The meta-analysis revealed no significant difference in the KSS between the FB and MB groups (WMD=0.80; 95% CI, −1.36–2.97; P=0.47), and heterogeneity tests indicated minimal interstudy heterogeneity (P=0.98 and I^2^=0%; fixed-effect analysis; Fig. 5). In addition, a fixed-effect model of the pooled data demonstrated that there was no statistical difference in the HSS knee score between the two groups (WMD=−0.65; 95% CI, −3.07-1.77; P=0.60). The study conducted by Jolles *et al* (26) was the sole study to assess WOMAC scores, and revealed that there was no significant difference between the groups (P=0.94).

**Figure 6.**
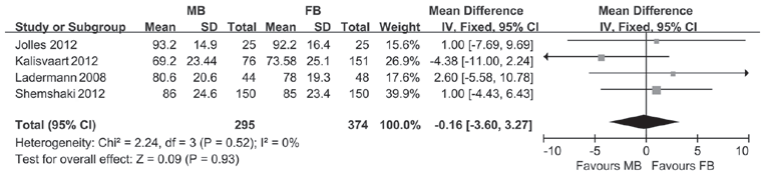
Forest plot comparing the Knee Society functional outcome scores of mobile-bearing (MB) and fixed-bearing (FB) implants. CI, confidence interval.

Four of the trials stated the KSS-function outcomes (25–27,29). There was no indication of statistical heterogeneity between the trials (P=0.52 and I^2^=0%), and a fixed-effect model of the pooled data revealed no significant difference in the KSS-function outcomes between the FB and MB TKAs (WMD=−0.16; 95% CI, −3.60-3.27; P=0.93; Fig. 6).

#### Maximum knee flexion

There were three trials (551 knees) that stated the maximum knee flexion (25,27,31). The meta-analysis did not demonstrate a significant difference between the two groups (WMD=−0.52; 95% CI, −3.36-2.32; P=0.72), and there was minimal interstudy heterogeneity (P=0.47 and I^2^=0%; fixed-effect analysis).

#### Pain

The meta-analysis of the visual analog scales (VAS) of pain in two of the trials indicated no significant difference between the MB and FB TKAs (WMD=−0.02; 95% CI, −0.62-0.59; P=0.96), with minimal interstudy heterogeneity (P=0.66 and I^2^=0%; fixed-effect analysis) (25,26).

#### Patient preference

Only one trial revealed the patients’ preferences with regard to their knee replacements. In the study by Matsuda *et al* (17), 13 patients underwent bilateral arthroplasty, where one knee was replaced with an FB prosthesis and the other with an MB prosthesis. In these patients, one patient favored the knee with the FB prosthesis, two patients favored the MB prosthesis and the remaining ten patients stated that there was no difference between their knees.

#### Quality of life

Three methods were used to measure the quality of life in the selected studies. Shemshaki *et al* (29) evaluated short form 36 (SF-36) scores, Lädermann *et al* (25) assessed SF-12 scores and Jolles *et al* (26) used EuroQol-5D scores. No significant differences in the quality of life scores between the MB and FB TKAs were demonstrated in the studies (P>0.05).

#### Radiological outcomes

There were two trials (277 knees) that revealed the occurrence of nonprogressive radiolucent lines around the tibial or femoral components (26,27). The meta-analysis demonstrated that there was no significant difference between the FB and MB TKAs (RR=1.05; 95% CI, 0.50–2.18; P=0.90).

#### Survivorship

Three studies (24,27,28) revealed the Kaplan-Meier survival estimates for the prostheses; however, two of these studies (24,28) did not contain sufficient information for the meta-analysis. Kalisvaart *et al* (27) indicated that no difference was detected in implant durability or fixation between the FB and MB prostheses after five years; Kaplan-Meier analysis demonstrated five-year implant survival rates of 98.0 and 98.7% for the FB and MB groups, respectively, with revision surgery for any reason taken as the end point. Kim *et al* (28) reported that the Kaplan-Meier survival estimate revealed 99 and 100% survival rates of the prosthesis in the MB and FB groups, respectively, after five years (95% CI, 0.89–0.97), with all reasons for failure taken into account. Mahoney *et al* (24) indicated that survival was similar (P=0.351) between the MB and FB groups, with revision of any component for any reason taken as the end point [90.1% (95% CI, 84.1–93.9) and 94.2% (95% CI, 90.1–96.6) for the MB and FB groups, respectively).

#### Complications

Five of the studies provided data on postoperative complications, including aseptic loosening, infection, revision, patellar crepitus, complete wear of the polyethylene tibial bearing and knee stiffness (19,20,22,23,27). The meta-analysis did not identify any significant differences in the RRs between MB and FB TKAs, with respect to aseptic loosening, infection, revision, patellar crepitus or knee stiffness (Table II). Kim *et al* (31) reported two cases of complete wear of the polyethylene tibial bearing in the FB cohort, and one case of dislocation of the medial polyethylene tibial bearing in the MB cohort.

## Discussion

There has been a great interest in MB prostheses, due to their assumed reduction in polyethylene wear and component loosening, in comparison with FB prostheses (32–34). The current study was designed to determine whether this theoretical superiority has been translated into improved clinical outcomes at the mid- to long-term follow-up. The results of this review demonstrated that there was no significant difference with respect to clinical, functional or radiological outcomes, or complication rates between the FB and MB TKA designs. However, the review has revealed two factors to be considered.

The mean follow-up duration of the trials included in this study was 5 years, and only two trials (25,31) had a follow-up duration of >7 years. The expected advantages of the MB prosthesis, i.e. reduced wear and diminished implant loosening, may only become apparent following a long period of time. Therefore, trials with a longer follow-up duration are required before a definite conclusion may be reached. In addition, different MB implant designs were used in different trials. Although all the designs featured polyethylene-bearing mobility, the MB implants differed according to whether the posterior cruciate ligament was retained or sacrificed, or whether the implant was substituted with a different brand. It has been demonstrated that different types of implant elicit different effects on the ROM achieved following the TKA (35). It is possible that the variability amongst the different MB and FB prostheses may have adversely impacted the accuracy of the conclusion of the present study.

Polyethylene wear was a predominant reason for the development of MB designs. The aim of the MB design is to reduce the overall polyethylene wear through increased contact area and congruency, while minimizing the constraint and maintaining the normal knee motion. A study conducted by Parks *et al* (36) demonstrated that an undersurface stress existed between the MB undersurface of the polyethylene and the metal tray that was 40% of the uppersurface stress, and retrieval studies indicated that the MB inserts had an improved wear rate in comparison with the FB inserts (0.04 versus 0.07 mm/year, respectively) (37). However, Kelly *et al* (38) demonstrated that the MB surface was damaged to an extent similar to that of the FB surface, in a study of 48 retrieved MB TKAs. Kim *et al* (31) revealed that there were two cases that were revised due to complete wear of the polyethylene tibial bearing in the FB cohort; however, no wear rate was revealed in any of the trials included in the present study.

As mentioned previously, the MB TKAs may theoretically prolong the implant survival by reducing shear forces at the polyethylene interface, reducing contact forces and enabling the mobility of the bearing surface (39–41). However, in the current review, the three studies that revealed the Kaplan-Meier survival estimates did not demonstrate any significant differences between the MB and FB TKAs at the five-year follow-up. Certain studies revealed the survivorship for TKA as 91% at 14 years (42) and 92% at 15 years (43), with repeated surgery for any reason as the endpoint. Therefore, a sufficiently long-term follow-up is required in order to evaluate the survivorship of TKA.

Allocation concealment is one of the most important biases to minimize when establishing the quality of the evidence. Since only two of the nine trials clearly described the method of allocation concealment used, it is possible that allocation bias occurred in the other trials. The blinding of patients was accomplished in <50% of trials, and the evidence was indeterminate, as it related to functional assessment. However, the blinding of the clinical assessors was performed in the majority of the studies, which reduced the detection bias. Certain trials did not reveal important results, which diminished the ability of the present study to estimate the effectiveness of the treatments. The interpretation of the results of the studies was limited, as, according to the criteria for the judgement of the risk of bias, all the studies were at a high risk of bias.

In conclusion, the results of this meta-analysis suggest that MB and FB prostheses have no statistically significant differences in clinical or radiological outcomes, or in complication rates. In order to perform an improved assessment of the efficacy of MB implants, well-designed RCTs that compare FB and MB prostheses, and have a long-term follow-up, are required.

## Figures and Tables

**Table I.** Search strategies.

Database	Period of search	Search strategy
Medline (Pubmed)	1966-August 2012	(((((“Arthroplasty, Replacement, Knee”[Mesh])) OR (((knee)) AND (((((replacement*) OR arthroplast*) OR prosthe*) OR implant) OR endoprosthe*)))) AND (((((mobile bearing) OR mobile platform) OR rotating platform) OR meniscal bearing) OR gliding bearing)) AND ((randomized controlled trial[pt] OR controlled clinical trial[pt] OR randomized[tiab] OR placebo [tiab] OR clinical trials as topic[mesh:noexp] OR randomly [tiab] OR trial[ti]) NOT (animals[mh]NOT humans[mh]))
Cochrane Central Register of Controlled Trials in the Cochrane library	Issue 2, 2012	#1 MeSH descriptor Arthroplasty, Replacement, Knee explode all trees
		#2 (replacement*) or (arthroplast*) or (prosthe*) or (implant) or (endoprosthe*)
		#3 (knee)
		#4 (#2 AND #3)
		#5 (#1 AND #4)
		#6 (mobile bearing) or (mobile platform) or (rotating platform) or (meniscal bearing) or (gliding bearing)
		#7 (#1 AND #6)
Embase	1974-August 2012	‘knee arthroplasty’/exp OR ‘total knee replacement’/exp OR (replacement* OR arthroplast* OR prosthe* OR implant OR endoprosthe* AND knee) AND (mobile AND bearing OR (mobile AND platform) OR (rotating AND platform) OR (meniscal AND bearing) OR (gliding AND bearing)) AND (random* OR blind* OR placebo OR ‘meta analysis’)

**Table II.** Meta-analysis of the complications in fixed-bearing and mobile-bearing TKAs.

Complication	Studies assessed	Incidence		Relative Risk (95% CI)	Overall effect (P-value)	Heterogeneity	
		Mobile	Fixed			I^2^ (%)	χ^2^ (P-value)
Aseptic loosening	(19,22)	2/328	3/406	1.11 (0.21, 5.89)	0.90	44	0.18
Revision	(19,20,27)	25/412	17/419	1.48 (0.82, 2.67)	0.20	0	0.60
Infection	(19,20,22,27)	7/546	2/628	2.75 (0.81, 9.33)	0.11	0	0.85
Patellar crepitus	(22,23)	45/250	53/325	0.86 (0.61, 1.21)	0.39	0	0.55
Knee stiffness	(19,20,22)	9/372	7/454	1.56 (0.62, 3.94)	0.35	0	0.55

TKA, total knee arthroplasty; CI, confidence interval.
